# Combined Oral and Intravenous Immunization Stimulates Strong IgA Responses in Both Systemic and Mucosal Compartments

**DOI:** 10.1371/journal.pone.0168037

**Published:** 2016-12-09

**Authors:** Zhe Yang, Qing Zhao, Yun-An Gao, Wei Zhang

**Affiliations:** Department of Immunology, Institute of Basic Medical Sciences, Chinese Academy of Medical Sciences, School of Basic Medicine, Peking Union Medical College, Beijing, People’s Republic of China; Food and Drug Administration, UNITED STATES

## Abstract

To investigate the influence of immunization routes onIgG, IgA and IgM production in systemic and mucosal compartments, we immunized mice with keyhole limpet hemocyanin (KLH) via oral, intranasal (i.n.) or subcutaneous (s.c.) routes alone or combined with the intravenous (i.v.) route. We found that administering antigen intravenously could affect antibody production and formation of antibody secreting cells (ASCs) depending on the immunization route previously used. Combined oral/i.v. immunization but not s.c./i.v. immunization caused a great increase of IgA ASCs in the spleen and enhanced IgA production in the small intestine and serum. Combined i.n./i.v. immunization could also increase IgA ASCs in the spleen and enhance IgA production in serum but had no effect on IgA production in the small intestine. Oral/i.v. immunization caused increase of IgG ASCs in both the spleen and bone marrow. In comparison, combined i.n./i.v. and s.c./i.v. immunization could increase IgG ASCs in the spleen but not in bone marrow. Intravenous administration of KLH in mice that had been immunized via oral, i.n. or s.c. routes caused some increase of IgM ASCs in the spleen but not in bone marrow. In conclusion, combined oral and i.v. administration of an antigen can induce fast and strong immune responses, especially for IgA, in both systemic and mucosal compartments.

## Introduction

The effector sites of immunocytes against invading pathogens are limited in certain compartments in the body. For antibody secreting cells (ASCs), they are mainly located in the lamina propria of the mucosa, bone marrow, peripheral lymphatic tissue and inflammation sites [1, 2]. ASCs at different sites express antibodies with different isotype preferences. Serum antibodies are expressed by ASCs in peripheral lymphatic tissue and bone marrow. In the serum, IgG is the predominant antibody while IgA and IgM are less so and IgE is extremely little. Mucosal antibodies are mainly expressed by ASCs in the lamina propria of the mucosa. IgA is the major antibody isotype; IgM and IgG can also be expressed, but at lower levels [3]. Antibodies with different isotypes have different functions in the body. For example, mucosal IgA antibodies can be translocated onto the mucosal surface to block pathogen interactions with host cells. Serum IgG antibodies usually bind to antigens and form immune complexes, leading to complement activation and stimulation of phagocytes to overtake and degrade the antigens.

B cells need to be stimulated to differentiate into ASCs. The inductive sites and effector sites of ASCs are often separate. After stimulation, each ASC expresses a certain set of homing receptors, which leads the cell to migrate and reach its effector site [1]. An important consideration in vaccine design is to determine optimal routes to induce immune responses and allow immunocytes to function at appropriate sites. Researchers have found that although both oral and nasal immunization can elicit antigen-specific immune responses in mucosal tissues, the responses may not be at the same sites [4–9]. Oral immunization induces specific IgA production in the small intestines and the salivary and mammary glands but not in the female genital tract [10–12]. Nasal immunization can elicit strong immune responses in respiratory and cervicovaginal mucosa but fails to produce an effective immune response in the intestines [5, 6]. It has been noted that immunocytes activated at one mucosal site can disseminate immunity to remote mucosal tissues rather than to systemic sites, while immunocytes primed in systemic lymphoid tissues are mostly excluded from mucosal sites [4].

To stimulate efficient immune responses at demanded sites, researchers have tried using various administration routes, such as oral, nasal, rectal, genital, or tonsillar immunization [10–14].Researchers have also tried combined routes to stimulate protections at both systemic and mucosal compartments [15–20]. Several groups investigated systemic followed by mucosal routes of immunization [16, 17, 21, 22], and others focused on mucosal followed by systemic routes of immunization [8, 19, 20, 23, 24]. For example, researchers have used mucosal followed by systemic immunization against HIV infection and i.n. followed by intramuscular immunization to treat tuberculosis.However, the effectiveness of combined systemic and mucosal routes of immunization remains controversial [15]. In addition, little research has been focused on the effectiveness of mucosal immunization followed by i.v. administration of antigens, especially for IgA responses.

In this work, we used a model antigen, keyhole limpet hemocyanin (KLH), to study antibody responses to an antigen administered through different routes with or without receiving antigen intravenously. We found that combined oral/i.v. routes of immunization caused great increase in IgA response in the spleen and small intestine. Combined i.n./i.v. routes also caused increased IgA response in the spleen but not in the small intestine. Combined s.c./i.v. immunization had no effect on IgA production. Combined s.c./i.v. immunization could enhance specific IgG and IgM production in the spleen but not in bone marrow or the small intestine. Thus, compared with single oral, i.n. or s.c. routes, i.v. administration of an antigen may enhance antibody response depending on the immunization route previously used on the mice in the study. In addition, i.v. administration of antigen had significant impact on IgA production in systemic compartments.

## Materials and Methods

### Ethics statement

All experimental manipulations were undertaken in accordance with the Institutional Guidelines for the Care and Use of Laboratory Animals, Institute of Basic Medical Sciences, Chinese Academy of Medical Sciences (Beijing, China).The protocol was approved by the ethics committee of Basic Medical Sciences, Chinese Academy of Medical Sciences (Permit Number: ACUC-A02-2013-035). The work was performed under the principle of the 3Rs (replacement, reduction and refinement), and efforts were made to minimize animal suffering.

### Animals

BALB/c mice (female, 6–8 weeks old with body weight 17±3 grams) were used for mucosal immunizations and BALB/c mice (female, 4–6 weeks old with body weight 14±2 grams) were used for s.c. immunizations. The mice were purchased from the Institute of Laboratory Animal Sciences, Chinese Academy of Medical Sciences (Beijing, China). All mice were kept in a specific pathogen-free facilityin the Experimental Animal Center, Institute of Basic Medical Sciences, Chinese Academy of Medical Sciences (Beijing, China). Mice were kept under a 12 h light-dark cycle with food and water *ad libitum*. To minimize suffering of the mice, we performed the oral, i.n., s.c. and i.v. administrations with the minimum dose and frequency needed for effective response. The health of the mice was monitored daily, and there were no unexpected deaths during the experiment. Sacrifice of mice was performed by cervical dislocation.

### Antibodies and reagents

HRP-conjugated rat anti-mouse IgA was purchased from Southern Biotech (Birmingham, AL, USA). HRP-conjugated goat anti-mouse IgG and HRP-conjugated goat anti-mouse IgM were purchased from Sigma-Aldrich Co. (St. Louis, MO, USA). HRP-conjugated rat anti-human fibrinogen as an isotype control antibody was purchased from Absea (Beijing, China). Keyhole limpet hemocyanin (KLH) was purchased from Thermo Fisher Scientific (Waltham, MA, USA). Cholera toxin B subunit from *Vibrio cholerae* (CTB) was purchased from Sigma-Aldrich. Freund’s complete adjuvant(CFA) and Freund’s incomplete adjuvant(IFA) were purchased from Pierce Protein Biology (Rockford, IL, USA).

### Immunization

**Fig 1:**
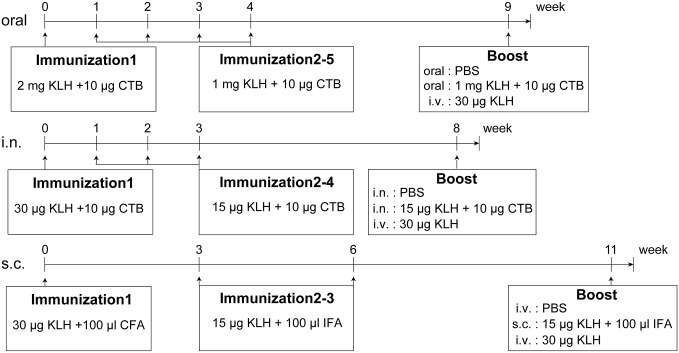
Vaccination schedule using different routes. BALB/c mice were immunized with KLH orally, intranasally or subcutaneously. The oral group was first immunized with 2 mg KLH and 10 μg CTB. Four boost immunizations were performed once a week with 1 mg KLH and 10 μg CTB. The i.n. group was first immunized with 30 μg KLH and 10 μg CTB intranasally. Three boost immunizations were performed once a week with 15 μg KLH and 10 μg CTB. The s.c. group was first injected with 30 μg KLH mixed with 100 μl CFA. Two boost immunizations were performed using 15 μg KLH mixed with 100 μl IFA at three-week intervals. Three days before sacrificing, mice were administered with KLH or PBS mucosally, subcutaneously or intravenously.

Mice were divided into oral, i.n. and s.c. groups. The oral group and the i.n. group had 24 mice each, while the s.c. group had 21 mice. The immunization schedule is shown in Fig 1. Mice in the oral group were first immunized by intragastric administration with 2 mg KLH and 10 μg CTB in PBS. Four boost immunizations were performed once a week with 1 mg KLH and 10 μg CTB. Five weeks after the last immunization, eight mice were administered intragastrically with PBS only, eight mice were administered intragastrically with1 mg KLH and 10 μg CTB, and eight mice were injected intravenously with 30 μg KLH. Mice in the i.n. group were first immunized with 30 μg KLH and 10 μg CTB intranasally. Three boost immunizations were performed once per week with 15 μg KLH and 10 μg CTB. Five weeks after the last immunization, eight mice were administered intranasally with PBS only, eight mice were administered intranasally with 15 μg KLH and 10 μg CTB, and eight mice were injected intravenously with 30 μg KLH. Mice in the s.c. group were first injected with 100 μl (30 μg) KLH mixed with 100 μl CFA. Two boost immunizations were performed using 100 μl (15 μg) KLH mixed with 100 μl IFA at three-week intervals. Five weeks after the last immunization, eight mice were injected intravenously with PBS only, five mice wereadministered subcutaneously with 100 μl (15 μg) KLH mixed with 100 μl IFAand eight mice were injected intravenously with30 μg KLH.

### Enzyme-linked immunosorbent assay (ELISA)

Microtiter plates (96-well) were coated with 2 μg/ml KLH at 4°C overnight in 15 mM Na_2_CO_3_ and 35 mM NaHCO_3_, pH 9.6. After being washed with PBS containing 0.05% Tween-20 (PBST), the plates were blocked with PBST containing 2% BSA for 1 h at room temperature. Serum and feces samples were collected before primary immunization or 7 days after each immunization. 100 mg of feces were soaked in 1 ml PBS at room temperature for 10 h and the supernatants were collected as feces extraction. 100 μl/well of the serum (serially 1:2 diluted from 1:1000 for IgG, 1:500 for IgA and IgM) in PBST containing 2% BSA or 100 μl/well of the feces extraction were added and incubated for 2 h at room temperature. After being washed with PBST, HRP-conjugated goat anti-mouse IgG, HRP-conjugated rat anti-mouse IgAor HRP-conjugated goat anti-mouse IgM was added. After incubation at room temperature for 1 h, the plates were washed with PBST, the substrate ABTS (AMERCO, Solon, OH, USA) was added, and the OD values were read at 405 nm.

### Enzyme-linked immunospot (ELISPOT)assay

MultiScreen IP filter plates (96-well) (Millipore, Billerica, MA, USA) were coated with 0.5 μg/well KLH in PBS at 4°C overnight. After being washed with PBS, the plates were blocked with 200 μl/well of RPMI 1640 medium containing 10% FCS at 37°C in a CO_2_ incubator. Mice were sacrificed 3 days after the last administration of KLH or PBS. Spleen, bone marrow and blood cells were collected and suspended in RPMI 1640 medium containing 10% FCS. Red blood cells were lysed using buffer containing 0.68% NH_4_Cl in 20 mM Tris-HCl, pH 7.2. Spleen cells (5×10^6^, 10^6^, 5×10^5^, 10^5^ and 5×10^4^), bone marrow cells (5×10^6^, 10^6^, 5×10^5^, 10^5^ and 5×10^4^) and peripheral white blood cells (2×10^5^) suspended in 100 μl RPMI 1640 medium containing 10% FCS were added to each well and cultured at 37°C for 18 h in a CO_2_ incubator. Then the cells were removed, the plates were washed once with cold distilled water and three times with PBST, and 100 μl/well of the HRP-conjugated goat anti-mouse IgG, HRP-conjugated rat anti-mouse IgA or HRP-conjugated goat anti-mouse IgM was added. After incubation at 37°C for 1 h, the plates were washed with PBST, and the AEC coloring system (DAKEWE Biotech, Beijing, China) was used for color development. After incubation at room temperature for 20 min, the reaction was stopped by washing with distilled water, and the plates were left to dry until counted. The spots were read and analyzed using CTL ImmunoSpot analyzers (Cellular Technology, Shaker Heights, OH, USA).

### Immunohistochemical (IHC) staining

Spleens and small intestines were fixed in 10% buffered formalin and embedded in paraffin. Thenthey were sectioned into 3-μm slices, which were placed on slides. After deparaffinization and hydration, antigens were retrieved in 0.01 M sodium citrate, pH 6.0, by microwave. After being treated with 3% H_2_O_2_ in methanol to extinguish the endogenous peroxidase activity, the slides were blocked for 20 min with 8% normal goat serum (ZSGB-BIO, Beijing, China) and incubated with HRP-conjugated rat anti-mouse IgA and HRP-conjugated rat anti-human fibrinogen at 37°C for 1 h. The color was developed using DAB (ZSGB-BIO, Beijing, China) as a substrate. The slides were then counterstained with hematoxylin (ZSGB-BIO, Beijing, China).

### Hematoxylin-eosin (HE) staining

Spleens and small intestines were fixed in 10% buffered formalin and embedded in paraffin. Thenthey were sectioned into 3-μm slices, which were placed on slides. After deparaffinization and hydration, the slides were stained with hematoxylin and eosin (ZSGB-BIO, Beijing, China).

### Data analysis

Data analysis was performed using the GraphPad Prism software (Version 5.01). We used Student *t* test to analyze differences. *P*< 0.05 was considered significant. Data are presented as mean ± SEM.

## Results

### ELISPOT assays for KLH-specific ASCs induced by different immunization routes

To compare Ag-specific immune responses after immunization via oral, i.n. and s.c. routes alone or in combination with the i.v. route, we examined levels of KLH-specific ASCs in the spleen, bone marrow and blood. Mice were primed and boosted with KLH via oral, i.n. or s.c. routes. Three days before sacrifice, they were given KLH or PBS via oral, i.n., s.c. or i.v. routes (Fig 1). ELISPOT was performed to analyze KLH-specific IgG, IgA and IgM ASCs from the spleen, bone marrow and blood. Positive spots from mice that were immunized with KLH and finally given PBS were counted as the basal levels of Ag-specific ASCs. Compared with the basal levels, the effectiveness of the finally administered KLH via different routes could be determined.

**Fig 2:**
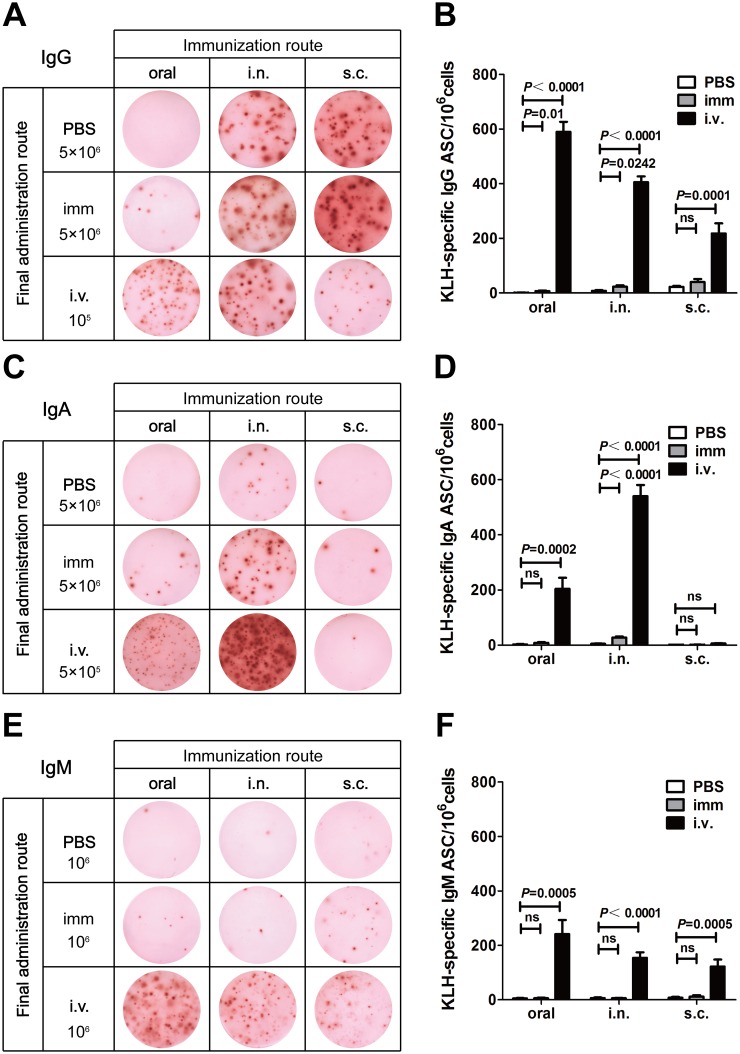
ELISPOT analysis for KLH-specific ASCs in the spleen. BALB/c mice were immunized with KLH via oral, i.n. or s.c. routes. Three days before sacrificing, mice were administered with PBS via the previous immunization routesor administered with KLH either via the previous immunization routes (imm) or via the i.v. route. Then, spleen cells were analyzed by ELISPOT for KLH-specific cells (see Materials and Methods). (A) Representative results of eight experiments for IgG ASCs (The s.c./s.c. group were of five experiments). 5×10^6^ cells were added each well for the top and middle rows, and 10^5^ cells were added each well for the bottom row. (B) ELISPOT counts of KLH-specific IgG ASCs. (C) Representative results of eight experiments for IgA ASCs (The s.c./s.c. group were of five experiments). 5×10^6^ cells were added each well for the top and middle rows, and 5×10^5^ cells were added each well for the bottom row. (D) ELISPOT counts of KLH-specific IgA ASCs. (E) Representative results ofeight experiments for IgM ASCs (The s.c./s.c. group were of five experiments). 10^6^ cells were added each well. (F) ELISPOT counts of KLH-specific IgM ASCs. The results presented in (B), (D), and (F) are the mean ± SEM for eightmice per group (The s.c./s.c. group were five mice).*P*> 0.05 was treated as not significant (ns).

The ELISPOT results of spleen cells are shown in Fig 2. The basal levels of KLH-specific ASCs depended on the routes of immunization and on the antibody isotypes tested. Mice immunized via the oral route and finally given PBS had almost no KLH-specific IgG, IgA and IgM ASCs in their spleens. Mice immunized via the i.n. route showed modest amounts of IgG ASCs and a few IgA ASCs. Mice immunized via the s.c. route also had modest amounts of IgG ASCs but very few IgA and IgM ASCs (top rows of Fig 2A, 2C and 2E). When mice were finally boosted with KLH, the number of positive spots depended largely on the administration routes. For mice that received KLH via the same as previous immunization routes, i.e., oral, i.n. or s.c., the number of positive spots kept unchanged or increased only slightly compared to the spots at the basal levels (middle rows of Fig 2A, 2C and 2E, Fig 2B, 2D and 2F). In comparison, most of mice showed great increase of positive cells in their spleens when they were finally boosted with KLH intravenously (bottom rows of Fig 2A, 2C and 2E, Fig 2B, 2D and 2F). Mice that were immunized mucosally and final boosted intravenously had more KLH-specific ASCs increase than the mice that had been immunized subcutaneously. As there were too many IgG and IgA ASCs after i.v. treatment, we showed the results of 10^5^ slpeen cells for IgG ASCs detection and 5×10^5^ spleen cells for IgA ASCs detection in Fig 2A and 2C to make the spots more countable. As IgM ASCs did not increase dramatically, we still used 10^6^ spleen cells for detection. Fig 2B, 2D and 2F showed the quantitative changes. The folds of increase were more for IgG and IgA ASCs than IgM ASCs. There was an exception for mice that were immunized subcutaneously. They hardly had any KLH-specific IgA ASCs in their spleens even after final administration of KLH via the i.v. route (bottom rows of Fig 2C and 2D).

**Fig 3:**
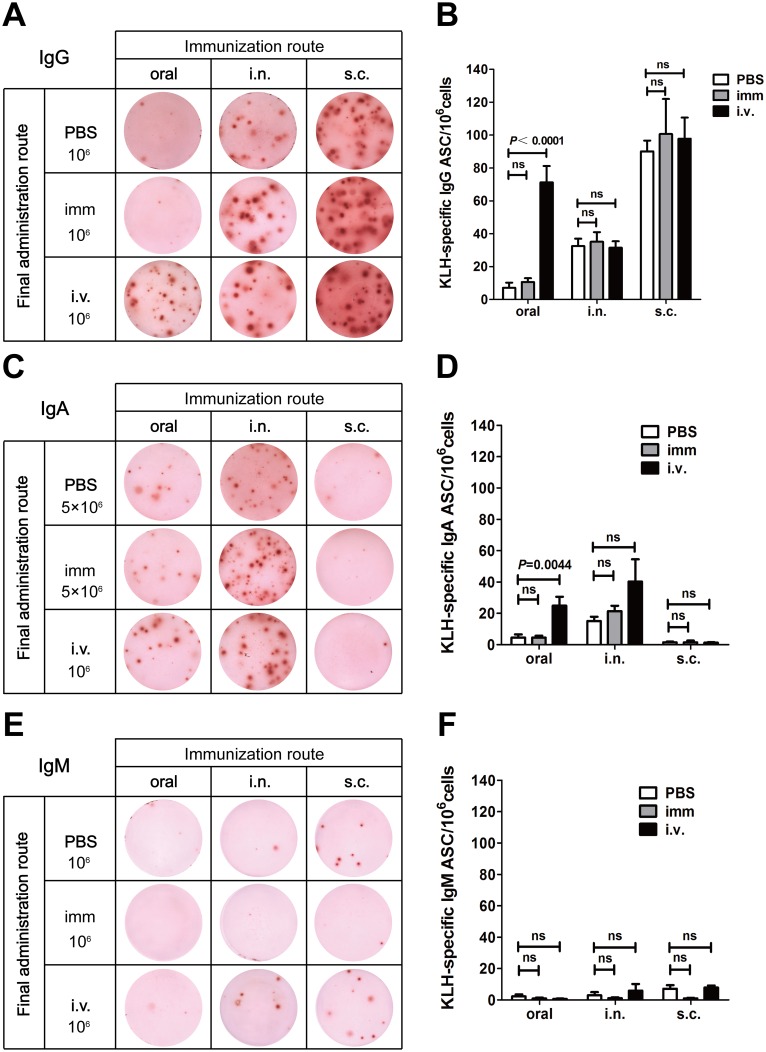
ELISPOT analysis for KLH-specific ASCs in bone marrow. BALB/c mice were immunized with KLH via oral, i.n. or s.c. routes. Three days before sacrificing, mice were administered with PBS via the previous immunization routesor administered with KLH either via the previous immunization routes (imm) or via the i.v. route. Then, bone marrow cells were analyzed by ELISPOT for KLH-specific cells (see Materials and Methods). (A) Representative results of eight experiments for IgG ASCs (The s.c./s.c. group were of five experiments). 10^6^ cells were added each well. (B) ELISPOT counts of KLH-specific IgG ASCs. (C) Representative results of eight experiments for IgA ASCs (The s.c./s.c. group were of five experiments). 5×10^6^ cells were added each well for the top and middle rows, and 10^6^ cells were added each well for the bottom row. (D) ELISPOT counts of KLH-specific IgA ASCs. (E) Representative results of eight experiments for IgM ASCs (The s.c./s.c. group were of five experiments). 10^6^ cells were added each well. (F) ELISPOT counts of KLH-specific IgM ASCs. The results presented in (B), (D), (F) are the mean ± SEM for eight mice per group (The s.c./s.c. group were five mice).*P*> 0.05 was treated as not significant (ns).

The ELISPOT results of bone marrow cells are shown in Fig 3. The patterns of the basal levels were similar to the spleen cells, i.e., mice immunized via the oral route andfinally given PBS had almost no KLH-specific IgG, IgA and IgM ASCs in their bone marrows. Mice immunized via the i.n. route showed modest amounts of IgG and IgA ASCs but hardly any IgM ASCs. Mice immunized via the s.c. route also had modest numbers of IgG ASCs but very few IgA and IgM ASCs (top rows of Fig 3A, 3C and 3E). When the mice were finally boosted with KLH via the same routes as previous immunization, the ELISPOT patterns were also similar to the spleen cells, i.e., administration of KLH did not cause substantial increase of ASCs compared to their basal levels (middle rows of Fig 3A, 3C and 3E, Fig 3B, 3D and 3F). When the mice were finally boosted with KLH via the i.v. route, however, the patterns were different from the spleen cells. Intravenous administration with KLH did not cause increase of Ag-specific ASCs in their bone marrows except to the mice that underwent oral immunization (bottom rows of Fig 3A, 3C and 3E, Fig 3B, 3D and 3F). Those mice had significant increase of IgG and IgA ASCs. However, the extent of increase was far less than the Ag-specific ASCs in spleens.

We also performed ELISPOT using 2×10^5^ peripheral white blood cells. However, there were too few positive cells to be detected (data not shown).

### IHC staining of IgA ASCs in spleens and small intestines from mice that underwent different immunization routes

**Fig 4:**
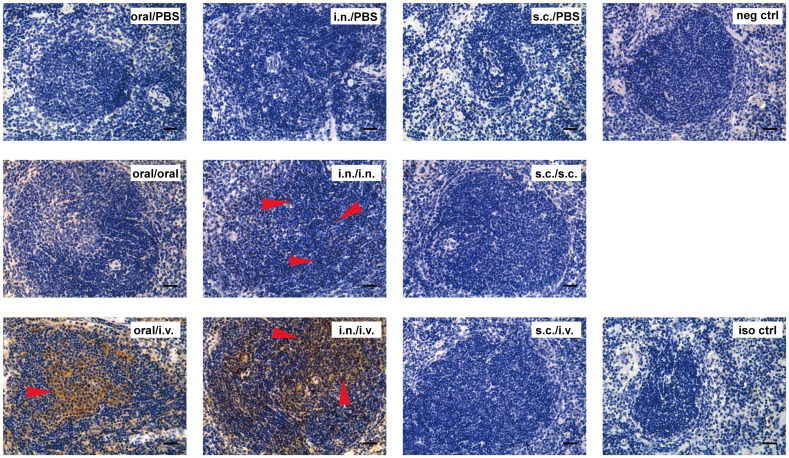
IHC analysis of IgA ASCs in the spleen. BALB/c mice were immunized with KLH via oral, i.n. or s.c. routes. Three days before sacrificing, mice were administered with KLH orally, intranasally, subcutaneously or intravenously. Spleens of mice were embedded in paraffin and sectioned into 3-μm slices and stained using anti-IgA antibody (see Materials and Methods). Top row, mice were immunized with KLH orally, intranasally or subcutaneously. Three days before sacrificing, the mice received PBS via oral, i.n. or i.v. routes. The negative control was from mice that did not receive inoculations. Middle row, mice were immunized with KLH and received a final immunization with KLH before sacrificing either orally, intranasally or subcutaneously. Bottom row, mice were immunized orally, intranasally or subcutaneously and administered a final immunization with KLH intravenously. The isotype control was stained using rat IgG1 anti-human fibrinogen antibody. Data are representative ofeightmice(The s.c./s.c. group in the middle row were five mice). Scale bar = 50 μm.

The above results showed that there was a significant increase of IgA ASCs in the spleens of mice that were immunized orally or intranasally and received the final antigen intravenously. To see localization of the IgA ASCs, we performed IHC analysis using paraffin sections of spleens from mice that had been immunized via different routes. The results are shown in Fig 4 and the corresponding results of HE staining are shown in S2 Fig. Mice that underwent oral immunization and received the final PBS or KLH orally did not show IgA ASCs in their spleens, but many IgA ASCs clustered together in the spleens of mice that received KLH intravenously (Fig 4, indicated by arrows in oral/i.v. group). In comparison, mice immunized intranasally did not show IgA ASCs when given the final PBS but did show dispersed staining after final i.n. administration of KLH and strong staining after i.v. administration of KLH(Fig 4, indicated by arrows in i.n./i.n. group and i.n./i.v. group). The locations of the IgA ASCs were spread out in all white pulps. The IHC staining still showed no IgA ASCs for mice that underwent s.c. immunization and i.v. administration of KLH.

**Fig 5:**
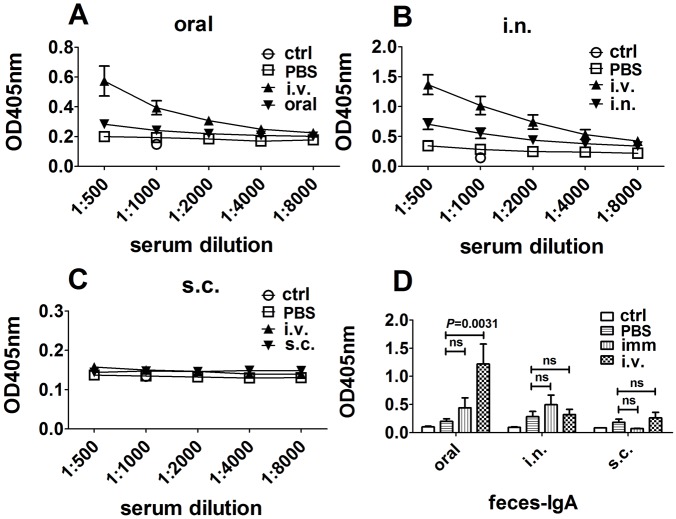
Levels of KLH-specific IgA antibodies in serum and feces after final administration of KLH. Oral and i.n. immunizations were performed four or five times at one-week intervals. Subcutaneous immunization was performed three times at three-week intervals. Three days before sacrificing, mice were received KLH via different routes. Serum and feces were collected, and the KLH-specific IgA antibodies were measured by ELISA in 96-well microtiter plates coated with KLH. Serum was 1 in 2 serially diluted (A, B, C). Feces were extracted by PBS (10 μl PBS per 1 mg feces) for 10 h and supernatants were collected (D). The anti-KLH IgA antibody was detected by HRP conjugated rat anti-mouse IgA. Group control means the mice did not receive inoculations. Group imm in D means the mice administered with KLH via the previous immunization routes (oral, i.n. or s.c.). Data shown are mean ± SEM for eightmice per group(The s.c./s.c. group in C and D were fivemice).

Although the above IHC staining was for all IgA ASCs, the fact that the IgA ASCs increased soon after final administration of KLH implied that the increased ASCs were induced by a specific antigen. To prove this, we measured serum anti-KLH IgA levels (Fig 5). As expected, final i.v. administration of the antigen caused an increase of anti-KLH IgA in serum in mice that were immunized orally and intranasally but had little effect on mice that were immunized subcutaneously (Fig 5A–5C). To verify the IHC result of the small intestine, we measured the feces extraction as well and found that mice immunized orally with final i.v. administration of KLH elicited more anti-KLH IgA (Fig 5D).

**Fig 6:**
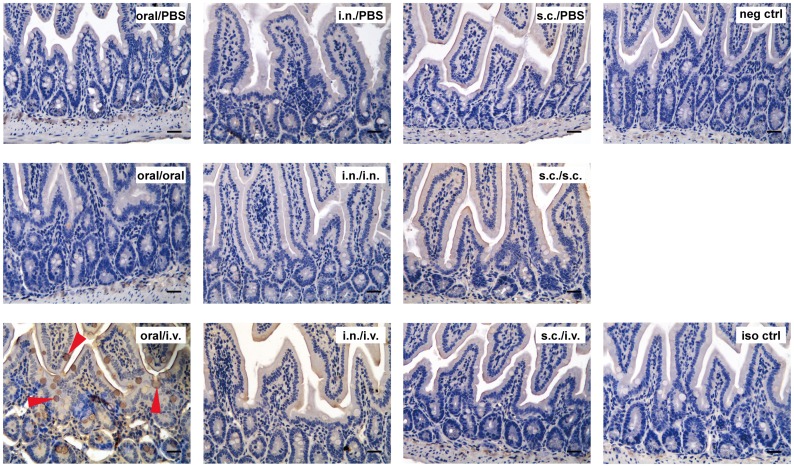
IHC analysis of IgA ASCs in the small intestines. BALB/c mice were immunized via oral, i.n. or s.c. routes. Three days before sacrificing, mice were administered with KLH orally, intranasally, subcutaneously or intravenously. The small intestines of mice were embedded in paraffin, sectioned into 3-μm slices and stained using an anti-IgA antibody (see Materials and Methods). Top row, mice were immunized with KLH orally, intranasally or subcutaneously. Three days before sacrificing, they were received PBS via oral, i.n. or i.v. routes. The negative control was from mice that did not receive inoculations. Middle row, mice were immunized with KLH. Three days before sacrificing, they were received KLH orally, intranasally or subcutaneously. Bottom row, mice were immunized orally, intranasally or subcutaneously and finally administered with KLH intravenously. The isotype control was stained using rat IgG1 anti-human fibrinogen antibody. Data are representative of eightmice(The s.c./s.c. group in the middle row were five mice). Scale bar = 50 μm.

We also performed IHC analysis for IgA in the small intestines. The results are shown in Fig 6 and the corresponding results of HE staining are shown in S3 Fig. Because IgA is constantly secreted and transported across the epithelium of the small intestines, it was not surprising that there was some background staining on the tissues. The interesting observation was that mice that were immunized orally and received the antigen intravenously had obvious IgA staining on the goblet cells (Fig 6, indicated by arrows), while none of the other mice showed the same phenomenon (Fig 6). This result implied that there was greater secretion of IgA into the lumen after i.v. administration of KLH in mice that were immunized orally. The ELISA result of feces also proved that the KLH-specific IgA was greatly increased in those mice but not in others (Fig 5D).

## Discussion

In this work, we compared efficiencies of different immunization routes on production of IgG, IgA, IgM and generation of Ag-specific ASCs. Our major discovery is that, compared to oral route only, final administration of antigen intravenously caused elevation of Ag-specific IgA in the small intestine and significant increase of IgA ASCs in the spleen. Intravenous administration of the antigen did not have the same effect on mice that had been immunized through the systemic (s.c.) route. A combination of i.n./i.v. routes also greatly increased IgA ASCs in the spleen but did not elevate secreted IgA in the small intestine. Intravenous administration of the antigen also affected the generation of IgG and IgM ASCs to various degrees.

It is known that the s.c. route of immunization can elicit immune responses in the systemic compartment [9]. We also found the serum IgA increase after oral or i.n. immunization (see Supporting Information, S1 Fig). Nevertheless, it was still a surprise for us to observe such a significant increase of IgA ASCs in the spleen after combined oral/i.v. or i.n./i.v. immunization. Although the mechanisms need to be studied further, we believe those IgA ASCs were originally stimulated in mucosa because mice needed to be immunized at mucosal sites first and mice that received the antigen through the systemic route (s.c.) did not produce more IgA ASCs after i.v. treatment. We suspect that the increased IgA ASCs in the spleen are related to the migration behaviors of the cells.

Migration of ASCs or their precursor plasmablasts in the body is tightly controlled. For mucosal IgA ASCs, B cells first encounter pathogens and switch to IgA^+^ cells at mucosal inductive sites such as Peyer’s patches. Then, the cells migrate to mesenteric lymph nodes, enter circulation via the thoracic duct and differentiate into IgA-bearing plasmablasts [25–27]. Subsequently, the majorities of the cells extravasate into mucosal effector sites, i.e., lamina propria, and become sessile IgA ASCs [28]. A few mucosal-activated IgA plasmablasts may enter bone marrow, and very few go into systemic lymphoid organs such as the spleen [29–31]. The selective homing migration is directed by the binding of organ-specific adhesion moleculesand chemokine receptors on blast cells to addressins and chemokines expressed in the target organs [1, 32–35]. IgA blasts rarely go to secondary lymphoid organs because most of these cells do not express L-selectin (CD62L), which is an adhesion molecular responsible for cell homing to secondary lymphoid organs [1, 34, 36]. We speculate that the splenic IgA cells after mucosal/i.v. treatment might be the rare L-selectin-expressing B cells originating at the mucosa and residing in the spleen as memory cells, or that they came from mucosal tissues because i.v. treatment caused a shift of their homing receptors from spleen-phobic to spleen-philic.

In contrast to the drastic increase of KLH-specific IgA ASCs in the spleens of mice that were immunized mucosally and received the final antigen intravenously, mice that were immunized subcutaneously and received the final antigen intravenously had few or no IgA ASCs in their spleens. Other researchers have also reported that s.c. injection induces systemic but little or no mucosal immunity [37, 38]. In other words, induction of mucosal immune responses is achieved only by the mucosal route and not by the systemic route. Antibody responses to combined s.c./i.v. immunization can only be limited in systemic compartments [9, 39–41]. In addition, we found high levels of IgG in the feces of mice that received subcutaneous immunization (S1 Fig), which indicated that IgG in secretions may be derived from the serum. Previous studies have proven that IgG may cross the intestinal epithelium by a special receptor, FcRn [42, 43].

Although both oral and i.n. immunization were mucosal routes and both their antibody responses were greatly enhanced by i.v. administration of the antigen, they differed in several ways. First, i.v. treatment had no effect on IgA production in the small intestine of mice that were previously immunized intranasally, which is not surprising as others also reported that i.n. immunization is unable to induce IgA responses in the small intestine because it does not induce α4β7 and CCR9 expression, which is necessary for the IgA ASCs to travel to the intestine [1, 5, 6, 44]. Second, we found that proliferated IgA ASCs in spleens clustered together after oral/i.v. immunization, whereas they spread out after i.n./i.v. treatment. The cause of the different distributions is not clear at present. We suspect that this is due to different expression levels of adhesion molecules and that i.n./i.v. routes made the cells more mobile. We also observed that without i.v. treatment, there were almost no IgG ASCs in the bone marrow of mice that had undergone only oral immunization. In contrast, there were more IgG ASCs in bone marrow after i.n. or s.c. immunization, which can be explained by the fact that i.n. and s.c. routes can induce α4β1 expression on these cells, which leads the cells to bone marrow, where the α4β1 ligand VCAM1 is expressed [2].

Serum IgA is the second most abundant immunoglobulin isotype in serum. Its production occurs in two distinct immunological compartments: mucosal and systemic. It has been said that misleading mucosal IgA ASCs to bone marrow may cause pathogenic conditions such as IgA nephropathy [45, 46]. In our study, we found that IgA produced in the mucosal compartment after either oral or intranasal immunization only caused Ag-specific IgA production in serum, although at lower levels than IgG (S1 Fig). The IgA levels were greatly increased after i.v. treatment, and we think that the induced IgA was mucosal in origin, as systemic compartments (s.c./i.v.) did not induce an increase in IgA. Nonetheless, we did not observe any deposition of IgA in the kidneys after i.v. administration of KLH to form IgA immune complexes in the blood (data not shown). This might be because the mice were sacrificed three days after receiving KLH, which may not have enough time to form IgA deposits on the kidneys.

In our work, we used Freund’s adjuvant for s.c. immunization and CTB as adjuvant for mucosal immunization. To our knowledge so far, the main function of Freund’s adjuvant is to enhance systemic immune responses which are mainly the IgG class [47]. CTB is a common mucosal adjuvant and can stimulate IgA responses to ingested antigens [48, 49]. The great increase of IgA ASCs in the spleen from the oral/i.v. or i.n./i.v. route should be partially contributed by CTB that evoked IgA immune responses first and the IgA ASCs then translocated to the spleen after i.v. boost.

In conclusion, combined oral/i.v. immunization can induce rapid IgA and IgG ASCs proliferation in the spleen and enhance IgA production in serum and in the small intestine. Combined i.n./i.v. immunization can also induce proliferation of IgA and IgG ASCs in the spleen but is unable to increase IgA secretion in the small intestine. Combined s.c./i.v. immunization can induce proliferation of IgG ASCs in the spleen but has no effect on proliferation of IgA ASCs. Our results have proven that under certain circumstances, mucosal IgA responses can extend to systemic compartments. As a result, the Ag-specific IgA is not only produced in mucosal areas but also in systemic tissues. However, the safety of the combined mucosal/i.v. immunization needs to be investigated.

## Supporting Information

S1 Fig Levels of KLH-specific antibodies in serum and feces. Oral and i.n. immunizations were performed four or five times at one-week intervals. Subcutaneous immunization was performed three times at three-week intervals. Before inoculation and seven days after last immunization, serum and feces were collected and the KLH-specific antibodies were measured by ELISA in 96-well microtiter plates coated with KLH. An anti-KLH antibody was detected by HRP-conjugated goat anti-mouse IgG (A and D), HRP-conjugated rat anti-mouse IgA (B and D) and HRP conjugated goat anti-mouse IgM (C and D). Group control means the mice before primary immunization. Data shown are mean ± SEM for twenty-fourmice per group(The s.c. group were twenty-one mice).
(TIF)

S2 Fig HEstaining of the spleen. The adjacent paraffin sections showed in Fig 4 were stained using hematoxylin and eosin (see Materials and Methods). Top row, mice were immunized with KLH orally, intranasally or subcutaneously. Three days before sacrificing, the mice received PBS via oral, i.n. or i.v. routes. The negative control was from mice that did not receive inoculations. Middle row, mice were immunized with KLH and received a final immunization with KLH before sacrificing either orally, intranasally or subcutaneously. Bottom row, mice were immunized orally, intranasally or subcutaneously and administered a final immunization with KLH intravenously. The isotype control was mice from oral/i.v. group. Scale bar = 50 μm.
(TIF)

S3 Fig HE staining of the small intestines. The adjacent paraffin sections showed in Fig 6 were stained using hematoxylin and eosin (see Materials and Methods). Top row, mice were immunized with KLH orally, intranasally or subcutaneously. Three days before sacrificing, they were received PBS via oral, i.n. or i.v. routes. The negative control was from mice that did not receive inoculations. Middle row, mice were immunized with KLH. Three days before sacrificing, they were received KLH orally, intranasally or subcutaneously. Bottom row, mice were immunized orally, intranasally or subcutaneously and finally administered with KLH intravenously. The isotype control was mice from oral/i.v. group. Scale bar = 50 μm.
(TIF)
