# Supplementary figures and images for: Combined Oral and Intravenous Immunization Stimulates Strong IgA Responses in Both Systemic and Mucosal Compartments
Source: PLoS One. 2016 Dec 9;11(12):e0168037. doi: 10.1371/journal.pone.0168037 (PMC5148103; doi:10.1371/journal.pone.0168037)

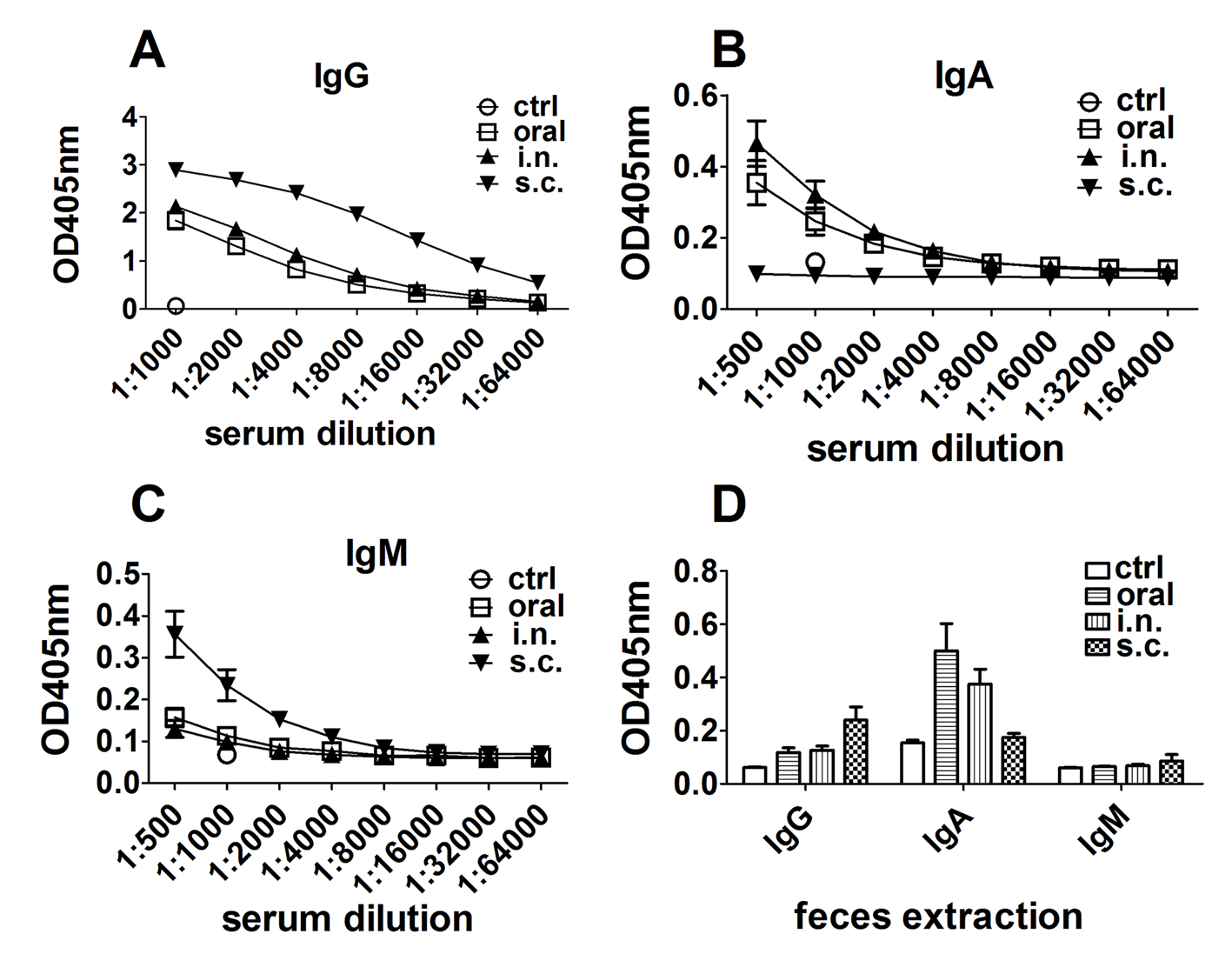

Supplement: S1 Fig — Oral and i.n. immunizations were performed four or five times at one-week intervals. Subcutaneous immunization was performed three times at three-week intervals. Before inoculation and seven days after last immunization, serum and feces were collected and the KLH-specific antibodies were measured by ELISA in 96-well microtiter plates coated with KLH. An anti-KLH antibody was detected by HRP-conjugated goat anti-mouse IgG (A and D), HRP-conjugated rat anti-mouse IgA (B and D) and HRP conjugated goat anti-mouse IgM (C and D). Group control means the mice before primary immunization. Data shown are mean ± SEM for twenty-fourmice per group(The s.c. group were twenty-one mice). (TIF) [file pone.0168037.s001.tif]

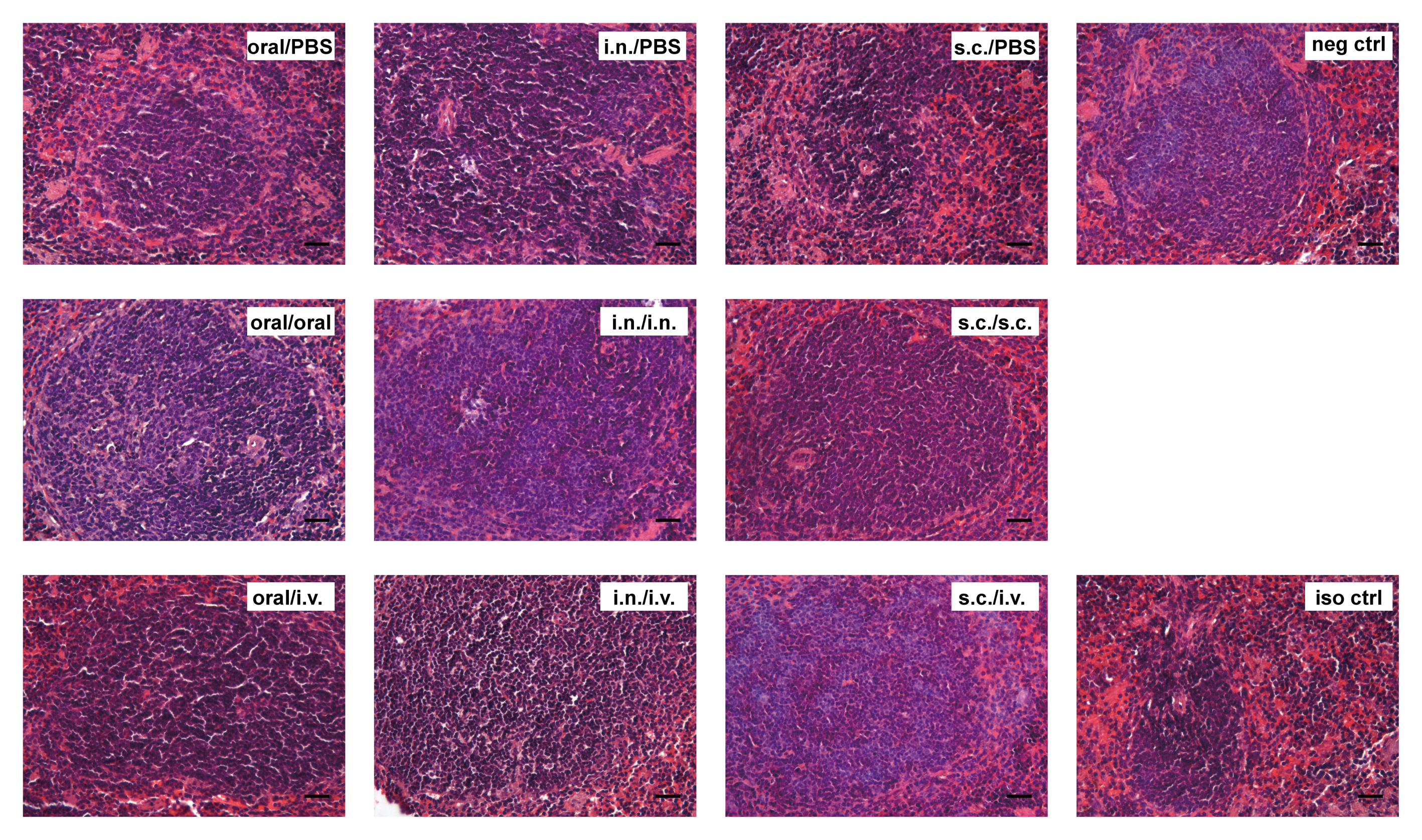

Supplement: S2 Fig — The adjacent paraffin sections showed in Fig 4 were stained using hematoxylin and eosin (see Materials and Methods). Top row, mice were immunized with KLH orally, intranasally or subcutaneously. Three days before sacrificing, the mice received PBS via oral, i.n. or i.v. routes. The negative control was from mice that did not receive inoculations. Middle row, mice were immunized with KLH and received a final immunization with KLH before sacrificing either orally, intranasally or subcutaneously. Bottom row, mice were immunized orally, intranasally or subcutaneously and administered a final immunization with KLH intravenously. The isotype control was mice from oral/i.v. group. Scale bar = 50 μm. (TIF) [file pone.0168037.s002.tif]

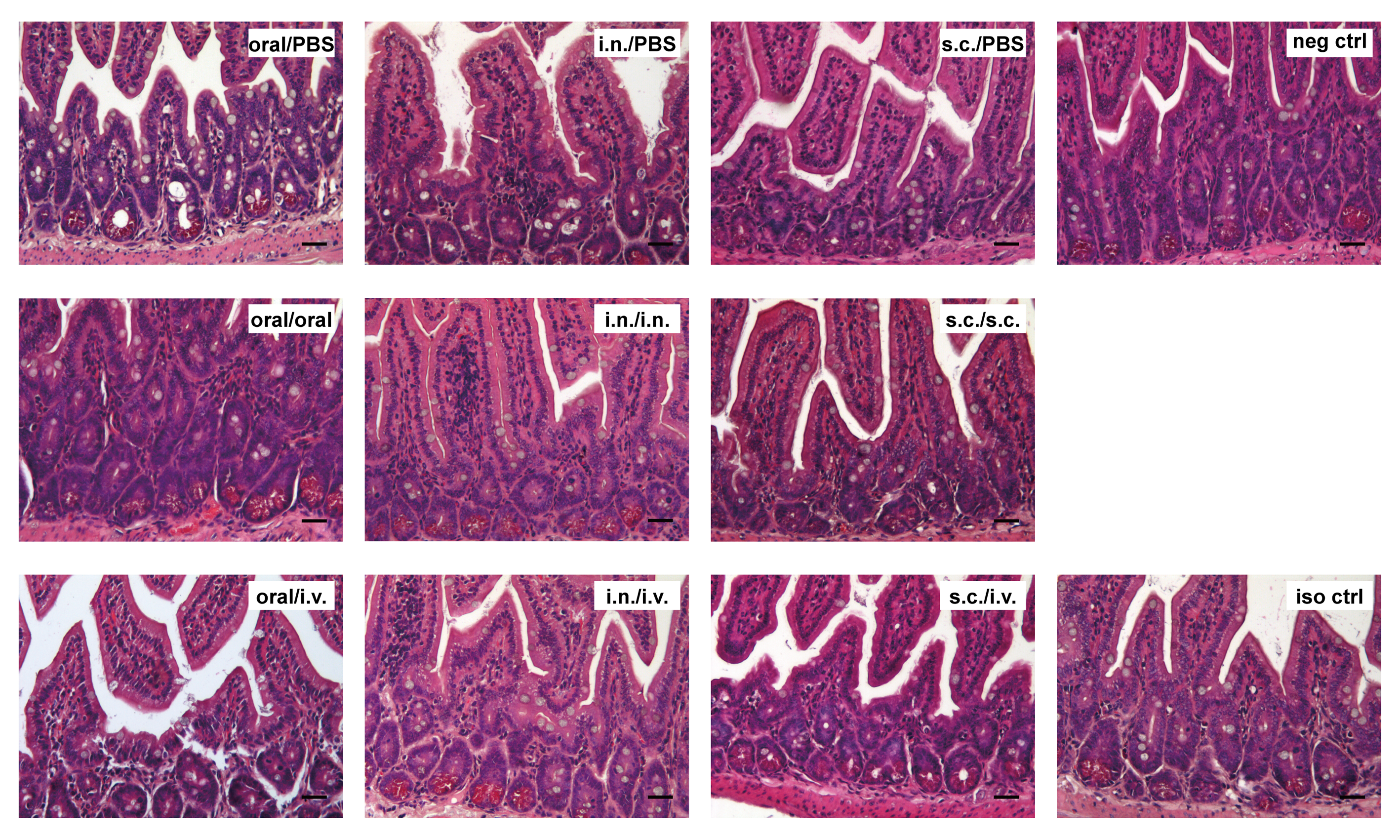

Supplement: S3 Fig — The adjacent paraffin sections showed in Fig 6 were stained using hematoxylin and eosin (see Materials and Methods). Top row, mice were immunized with KLH orally, intranasally or subcutaneously. Three days before sacrificing, they were received PBS via oral, i.n. or i.v. routes. The negative control was from mice that did not receive inoculations. Middle row, mice were immunized with KLH. Three days before sacrificing, they were received KLH orally, intranasally or subcutaneously. Bottom row, mice were immunized orally, intranasally or subcutaneously and finally administered with KLH intravenously. The isotype control was mice from oral/i.v. group. Scale bar = 50 μm. (TIF) [file pone.0168037.s003.tif]
